# Supplementary material for: Causal Relationship between Aging and Anorexia Nervosa: A White-Matter-Microstructure-Mediated Mendelian Randomization Analysis
Source: Biomedicines. 2024 Aug 16;12(8):1874. doi: 10.3390/biomedicines12081874 (PMC11351342; doi:10.3390/biomedicines12081874)
Supplement: Supplementary file 1 [file biomedicines-12-01874-s001.zip › Supplementary Figure/Figure S7/Single SNP plot of MR analysis for ubm-b-1924 to AN.pdf]

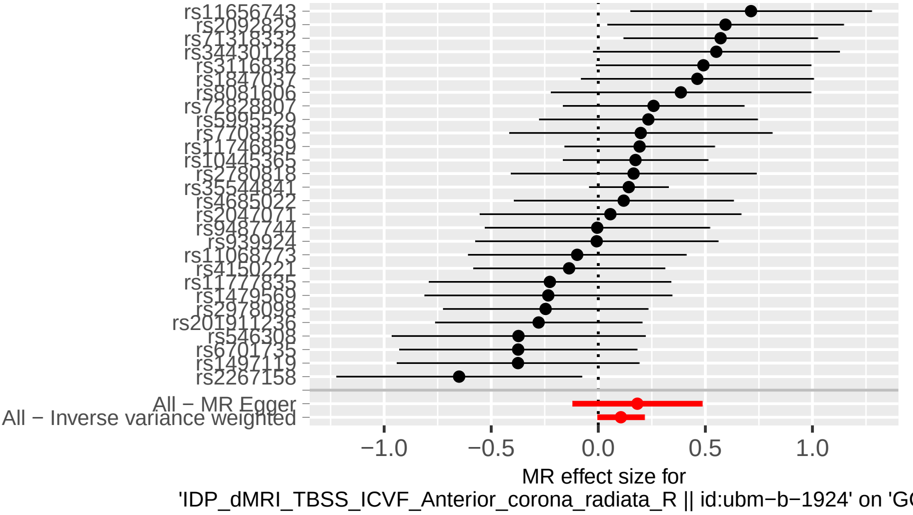
rs11656743
rs2092829
rs71318332
rs34430128
rs3116836
rs1847037
rs8081606
rs72828807
rs5995529
rs7708369
rs11746859
rs10445365
rs2780818
rs35544841
rs4685022
rs2047071
rs9487744
rs939924
rs11068773
rs4150221
rs11777835
rs1479569
rs2978098
rs201911236
rs546308
rs6701735
rs1497119
rs2267158
All − MR Egger
All − Inverse variance weighted
−1.0
−0.5
0.0
0.5
1.0
MR effect size for
'IDP_dMRI_TBSS_ICVF_Anterior_corona_radiata_R || id:ubm−b−1924' on 'G
